# Why do spatial abilities predict mathematical performance?

**DOI:** 10.1111/desc.12138

**Published:** 2014-01-11

**Authors:** Maria Grazia Tosto, Ken B. Hanscombe, Claire M.A. Haworth, Oliver S.P. Davis, Stephen A. Petrill, Philip S. Dale, Sergey Malykh, Robert Plomin, Yulia Kovas

**Affiliations:** ^1^ Department of PsychologyUniversity of YorkUK; ^2^ Laboratory for Cognitive Investigations and Behavioural GeneticsTomsk State UniversityRussia; ^3^ MRC SocialGenetic & Developmental Psychiatry CentreInstitute of PsychiatryKing's College LondonUK; ^4^ Department of PsychologyUniversity of WarwickUK; ^5^ UCL Genetics InstituteDepartment of GeneticsEvolution and EnvironmentUniversity College LondonUK; ^6^ Department of Human Development and Family ScienceOhio State UniversityUSA; ^7^ Department of Speech and Hearing SciencesUniversity of New MexicoUSA; ^8^ Department of PsychologyGoldsmith's College LondonUK

## Abstract

Spatial ability predicts performance in mathematics and eventual expertise in science, technology and engineering. Spatial skills have also been shown to rely on neuronal networks partially shared with mathematics. Understanding the nature of this association can inform educational practices and intervention for mathematical underperformance. Using data on two aspects of spatial ability and three domains of mathematical ability from 4174 pairs of 12‐year‐old twins, we examined the relative genetic and environmental contributions to variation in spatial ability and to its relationship with different aspects of mathematics. Environmental effects explained most of the variation in spatial ability (~70%) and in mathematical ability (~60%) at this age, and the effects were the same for boys and girls. Genetic factors explained about 60% of the observed relationship between spatial ability and mathematics, with a substantial portion of the relationship explained by common environmental influences (26% and 14% by shared and non‐shared environments respectively). These findings call for further research aimed at identifying specific environmental mediators of the spatial–mathematics relationship.

**Research highlights:**

- About a third of the variation in spatial ability at age 12 is explained by genetic factors; a little less than half of the variation in mathematics at this age is genetic.
- We find no sex differences in the genetic and environmental influences (either in magnitude or type) on mathematical and spatial variation at age 12.
- The observed overlap between spatial ability and mathematics is substantial (*r *>* *.40). Approximately 60% of this overlap is explained by common genetic effects, with 40% of the overlap due to environmental experience.

## Introduction

Individual differences in spatial and mathematical abilities are correlated (~.5, e.g. Hegarty & Kozhevnikov, 1999), and rely on partly overlapping neural networks (Hubbard, Piazza, Pinel & Dehaene, 2005). Spatial ability at age 18 moderately correlates with raw SAT (Scholastic Assessment Test) mathematics scores, and remains a significant predictor of mathematical ability after controlling for general intelligence, processing speed and working memory (Rohde & Thompson, 2007). Greater spatial ability at age 13 is associated with preference for mathematics‐related subjects at age 18;with choice of college major in Science, Technology, Engineering, or Mathematics (STEM), and with eventual expertise in STEM domains (e.g. Wai, Lubinski & Benbow, 2009).

Little is known about the aetiology of the associations between spatial abilities and mathematics. The only genetically sensitive study to date suggested that the moderate (.32) correlation between mathematical and spatial ability was largely explained by shared genetic effects. However, a small sample (*N *=* *278 twin pairs) and a wide age range (6–12 years) meant that the study was underpowered (Thompson, Detterman & Plomin, 1991).

Studies, mainly involving elementary–middle school students, suggest that at a cognitive level, several mechanisms are likely to underlie the space–mathematics association (e.g. Hegarty & Kozhevnikov, 1999). Research suggests that we think about numbers as organized in space along a mental ‘number line’ (Dehaene, Bossini & Giraux, 1993; Ito & Hatta, 2004), and that this mapping is independent from formal mathematical instruction (de Hevia & Spelke, 2009). Performance on a number line task correlates with later mathematical performance, suggesting that precision of symbolic number representation may bootstrap further mathematical learning (e.g. Siegler & Opfer, 2003). An observed correlation between performance on a 3‐D mental rotation task and mathematical word problem solving further supports the importance of spatial ability in mathematical learning (Johnson, 1984; van Garderen, 2006). Mathematical relations may be mentally spatially represented, such as the translation of word problems into equations (Geary, 1995). Moreover, representation and decoding of complex mathematical ideas may rely on spatial ability (Phillips, Norris & Macnab, 2010; Shoresh & Wong, 2012; Tufte, 2001).

Recent research has begun to identify brain mechanisms involved in the number–space cognitive processes. Brain damage resulting in unilateral neglect produces deficits in mental imagery and disrupts the ability to think of numbers in spatial terms, along a mental number line (Zorzi, Priftis & Umiltá, 2002). Differences in brain activation have been found during the mental rotation task between typically developing and mathematically gifted children (O'Boyle, Cunnington, Silk, Vaughan, Jackson, Syngeniotis & Egan, 2005). Children with developmental dyscalculia show structural deficits in brain areas involved in visuo‐spatial processing (Rykhlevskaia, Uddin, Kondos & Menon, 2009).

Genetically sensitive studies address the nature of these behavioural, cognitive, and neural associations. The present study is the first adequately powered investigation to evaluate the relative contribution of genetic and environmental factors to individual differences in spatial ability and to its relationship with different aspects of mathematics. By including same‐sex and opposite‐sex twins we also explored whether variation in spatial and mathematical abilities and the relationship between them is driven by the same genetic and environmental factors in males and females. Previous research found some evidence for an average male advantage in some spatial and mathematical tasks, but the results are inconsistent across studies (Casey, Nuttall, Pezaris & Benbow, 1995; Halpern, 2000; Levine, Huttenlocher, Taylor & Langrock, 1999; Astur, Tropp, Sava, Constable & Markus, 2004). Differences in the patterns of brain activation between males and females during spatial tasks have also been shown (Hugdahl, Thomsen & Ersland, 2006). However, the twin method explores the sources of individual variation, which can be unrelated to those of average sex differences.

## Method

### Sample

We assessed spatial ability and mathematics in a sample of twins drawn from the population‐based Twins Early Development Study (TEDS), with a mean age of 11.56 years (*SD* = 0.69).We had data for at least one twin in 4601 twin pairs (1663 MZ, 2938 DZ) for spatial ability and mathematics; of these, 4174 complete pairs (1539 MZ, 2635 DZ) provided data on all measures. The study identified from birth records all the twins born in England and Wales in 1994, 1995, and 1996 (Haworth, Davis & Plomin, 2013). More than 10,000 pairs of twins were recruited to the longitudinal study. Since the first contact with the twins' families, the TEDS sample remains representative of the United Kingdom (UK) general population. A validated parent‐rated instrument, with 95% accuracy when compared to zygosity established from DNA markers, was used to assign zygosity (Price, Freeman, Craig, Petrill, Ebersole & Plomin, 2000); uncertainties were followed up with DNA marker testing.

### Measures

**Figure 1:**
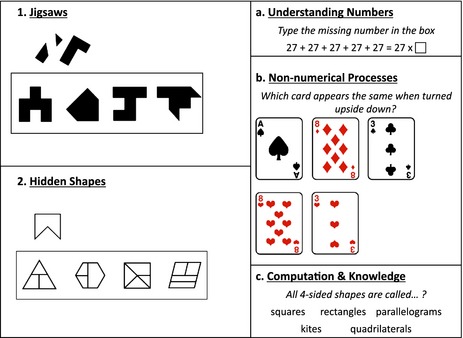
Spatial ability tests: 1. Jigsaws – which of four shapes is the assembly of the given set of smaller pieces, 2. Hidden shapes – in which of four complex patterns is the given polygon embedded; Mathematics subtests: a. application of numeric and algebraic processes, b. non‐numerical problems, c. recollection of mathematical facts and terminology.

Data for this study were collected using a web‐based battery of tests. Details of Internet testing and its validation can be found in Haworth *et al*. (Haworth, Harlaar, Kovas, Davis, Oliver, Hayiou‐Thomas, Frances, Busfield, McMillan, Dale & Plomin, 2007). Cronbach's alphas (α) reported below are based on the present sample. See Figure 1 for an example of each of the spatial ability and mathematics tests.

#### Spatial ability

Spatial ability was assessed by the Jigsaws and Hidden Shapes tests, drawn from the National Foundation for Educational Research Spatial Reasoning 8–14 series (Smith & Lord, 2002). The two tests require reasoning about the properties of shapes and their relationship in addition to the ability to visualize shapes and mentally manipulate them according to precise rules.

*Jigsaws* (28 items, α  =  0.74) was a multiple‐choice test assessing the ability to identify which of four shapes is the assembly of the given set of smaller shapes.

*Hidden Shapes* (27 items, α  =  0.87) was a multiple‐choice test assessing the ability to identify in which of four complex patterns a given polygon was embedded.

**Figure 2:**
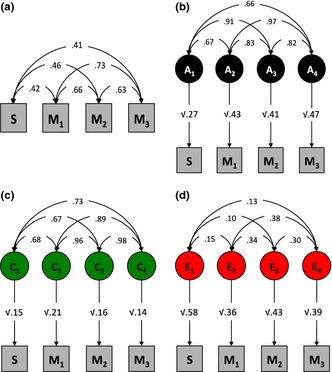
(a) Phenotypic (observed), (b) genetic (A), (c) shared (C), and (d) non‐shared (E) environmental correlations between spatial ability (S) and Understanding Numbers (M1), Non‐numerical Processes (M2), and Computation and Knowledge (M3).

Our two spatial tests correlated moderately with each other (*r *=* *0.34, *p *<* *.01) and both had similar correlations with the three mathematical components: the correlations of Hidden Shapes were .39 with Understanding Numbers (M1), .42 with Non‐numerical Processes (M2), .37 with Computation and Knowledge (M3). Correlations of Jigsaws were .30 with Understanding Numbers, .35 with Non‐numerical Processes, and .30 with Computation and Knowledge. The composite measure of spatial ability (S) correlated on average .43 with each of the three mathematics subtests (see Figure 2a for these phenotypic correlations).

Both spatial tests were normalized using a Van der Waerden rank transformation (Lehmann, 1975), and their mean was used to index a general spatial ability. All further analyses reported here were conducted on this restandardized composite spatial ability measure. The analyses conducted on the two measures separately yielded similar results (available in Supplementary Online Material, Figures S1, S2, S3 and S4).

#### Mathematical ability

Mathematics was assessed with a web‐based battery closely linked to the UK National Curriculum requirements, with items drawn from the nferNelson Mathematics age 5–14 series (nferNelson, 1994, 1999, 2001). The following three components of mathematics were assessed: *Understanding Numbers* (33 items, α  =  0.90) required an understanding of numeric and algebraic processes to be applied when solving problems; *Non‐numerical Processes* (25 items, α  =  0.87) required understanding of processes and concepts such as rotational and reflective symmetry, with no significant numerical content; *Computation and Knowledge* (37 items, α  =  0.93) required recollection of mathematical facts and terminology and the ability to perform straightforward calculations using well‐rehearsed techniques.

### The twin method

We used the twin method – a comparison of reared‐together identical (monozygotic) and non‐identical (dizygotic) twins – to partition the variance and covariance between spatial and mathematical ability into additive genetic (A), shared environmental (C), and non‐shared environmental (E) components (Boomsma, Busjahn & Peltonen, 2002; Plomin, DeFries, Knopik & Neiderhiser, 2013). The known genetic relatedness between monozygotic and dizygotic twins allows the decomposition of individual differences (or variation) in behaviour into A and C factors that make siblings reared together similar, and E factors that make them dissimilar.

### Structural equation modelling

Structural equation model fitting, with full information maximum likelihood estimation, provides a comprehensive way to estimate genetic and environmental variance components. We used the structural equation modelling package OpenMx (Boker, Neale, Maes, Wilde, Spiegel, Brick, Spies, Estabrook, Kenny, Bates, Mehta & Fox, 2011) in the statistical computing environment R (www.R-project.org; R Development Core Team, 2011) to fit ACE models. The fit of a given model to the observed data is summarized by a likelihood statistic (−2lnL; minus 2 * log likelihood) and degrees of freedom (*df*; observations − number of estimated parameters). Comparison of models is achieved with a chi‐square (*χ*^2^) likelihood‐ratio test, with *χ*^2^ given by the difference in fit (Δ−2lnL), and *df* given by the difference in degrees of freedom (Δ*df*). We also present the Bayesian information criterion (BIC: − ln(N)*df; Raftery, 1995), which is a measure of model fit relative to parsimony with lower BIC values indicating a better fit. In large datasets, BIC is preferred as it allows one to choose the most parsimonious model by taking into account the number of observations (sample size).

### Genetic analyses

#### Univariate analyses

We first analysed our spatial ability composite and the three aspects of mathematics with univariate sex‐limited models. Sex‐limitation modelling provides a test for the presence of *quantitative* and *qualitative* sex differences, irrespective of the mean differences between males and females. *Quantitative sex differences* are a difference in magnitude of genetic or environmental effects. For example, although the same genes may influence the trait in males and females, the effect may be stronger in males. Estimating A, C, and E covariance components separately for males and females, then comparing the model fit to one in which the covariance components are constrained to be equal for males and females, provides a test for the presence of quantitative sex differences. *Qualitative sex differences* suggest that a (partially) different set of genetic or environmental influences contributes to individual differences in the trait for males and females. With opposite‐sex twins it is possible to measure qualitative sex differences. For the DZ opposite‐sex pairs, allowing the coefficient of genetic relatedness (or the coefficient of environmental relatedness) to differ from that of the DZ same‐sex pairs, and comparing the model fit to one in which the coefficients are constrained to be equal, provides a test for the presence of qualitative sex differences.

#### Multivariate analysis

Multivariate structural equation modelling with twin data is an extension of the univariate twin analyses: both the variation within, and the covariation between, traits is divided into genetic and environmental components. The variable‐specific and common genetic, shared and non‐shared environmental factors were estimated by maximum likelihood using the Cholesky decomposition analysis (Loehlin, 1996). The Cholesky procedure is conceptually similar to a hierarchical regression where the trait‐specific variance is explained by trait‐specific latent factors after the common variance has been accounted for by common factors. As the variance explained by the latent factors is determined by the order of the variables in the model, we modelled the data including the spatial variable last, in order to estimate genetic and environmental influences on spatial ability independent from mathematics. We further converted the initial Cholesky decomposition into a correlated factor solution, which provides two useful summary statistics of the relationship between any pair of variables: the correlation between latent components, and the proportion of the phenotypic correlation explained by each of the ACE components. These parameters are not affected by the ordering of the variables. The correlation between latent genetic components can be interpreted as the extent to which genes found to be associated with one trait are associated with the other; the proportion of the phenotypic correlation explained by genetic factors describes the extent to which the relationship is driven by genes.

## Results

Descriptive statistics and analyses of variance (ANOVA) by sex and zygosity are shown in Table 1. To preserve independence of data, these analyses were conducted on one randomly selected twin in each pair. For both spatial ability and mathematics, *R*^2^ for the ANOVA models indicates that both sex and zygosity account for no more than 1% of the variance. Same‐sex twins are perfectly correlated for age and sex, an association that could be misinterpreted as within‐family resemblance due to the shared environment. So, as is standard practice in the analysis of twin data, all subsequent analyses were performed on the residuals after correcting for the effects of age and sex (McGue & Bouchard, 1984).

**Table 1:** Means, standard deviations and analysis of variance by sex and zygosity

											ANOVA						
* *	All		MZ		DZ		Female		Male		Zyg.		Sex		Zyg.*Sex		Tot.
Measures	*M*	*SD*	*M*	*SD*	*M*	*SD*	*M*	*SD*	*M*	*SD*	*p*	*η²*	*p*	*η²*	*p*	*η²*	*R* [Link]
Jigsaws total (0–28)	16.82	3.47	16.71	3.40	16.88	3.51	16.66	3.38	16.92	3.52	.337	<.001	.039	.001	.915	<.001	.002
Hidden Shapes total (0–27)	15.67	5.05	15.51	4.90	15.77	5.14	15.30	4.95	15.92	5.10	.527	<.001	<.001	.003	.124	.001	.004
Spatial Ability*	0.00	0.82	−0.04	0.80	0.02	0.84	−0.06	0.80	0.04	0.84	.220	<.001	<.001	.003	.431	<.001	.004
Understanding Numbers (0–33)	22.73	5.54	22.52	5.43	23.03	5.55	22.22	5.49	23.25	5.48	.155	<.001	<.001	.007	.684	<.001	.009
Non‐numerical Processes (0–25)	15.98	4.77	15.88	4.71	16.13	4.88	15.71	4.79	16.25	4.83	.522	<.001	.001	.002	.990	<.001	.003
Computation & Knowledge (0–37)	28.29	6.55	28.13	6.54	28.49	6.66	27.92	6.54	28.64	6.65	.378	<.001	.001	.002	.444	<.001	.003
Math total**	0.02	0.89	−0.04	0.86	0.05	0.90	−0.08	0.86	0.08	0.90	.112	.001	<.001	.005	.763	<.001	.008

*Spatial Ability is the mean of the two standardized spatial tests (requiring a score on both); **Math total is the mean of the three standardized mathematics tests (requiring a score on two out of the three subtests); ANOVA based on a random selection of one twin from each pair

ANOVA = analysis of variance; MZ = monozygotic; DZ = dizygotic; Zyg. = zygosity; Zyg.*Sex = zygosity by sex interaction term; Tot. = total variance explained by the ANOVA full model; *M * =  mean; *SD* = standard deviation; *p * =  *p*‐value; *η²* = partial eta squared; *R*^2^ =  variance explained by zygosity, sex, and zygosity × sex interaction.

Intraclass correlation coefficients (ICC; Shrout & Fleiss, 1979) provide coefficients of twin similarity and, when these are separated by sex and zygosity, give a first indication of potential sex differences (supplementary Table S1). For example, doubling the difference between the MZ and DZ ICCs for each sex/zygosity group provides a first approximation of the genetic contribution to each trait. Univariate sex limitation modelling provides a formal test of sex differences. Results of the univariate sex limitation analyses (summarized in supplementary Table S2) suggested no significant quantitative or qualitative sex differences for mathematics and spatial ability. Therefore, data from males and females were combined for the multivariate analyses.

### What is the nature of the relationship between spatial ability and mathematics?

The path diagrams in Figure 2 are a visual summary of the correlated factor model. The path values are standardized so that squaring each path gives the relative contribution of each variance component. Thus, the relative genetic contribution (or *heritability*) of spatial ability was a modest 27%. The mathematics subtests were moderately heritable – average heritability of 44% (Figure 2b). The same logic is applied to the calculation of the environmental influences (Figure 2c and 2d).

**Figure 3:**
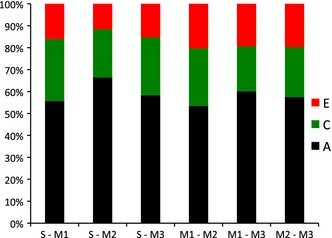
Proportion of the phenotypic correlation between Spatial Ability (S) and three mathematics subtests, Understanding Numbers (M1), Non‐numerical Processes (M2), and Computation and Knowledge (M3), explained by A, C, and E. A  =  additive genetic factors; C  =  shared environmental factors; E  =  non‐shared environmental factors.

In Figure 2, correlations between the latent genetic (2b) and environmental (2c and 2d) components are shown as double‐headed arrows. The total contribution of the genetic, shared environmental and non‐shared environmental factors to the phenotypic correlation between any two measured traits can be derived by multiplying each element in a path chain from one measured trait to the other, then summing these products for the A, C and E chains. Each phenotypic correlation can be broken down into fractions attributable to genetic, shared and non‐shared environmental factors by expressing each chain as a fraction of the sum of all three. For example, the genetic contribution to the phenotypic relationship between spatial ability and mathematics subtest Understanding Numbers, is given by: (√.27*.67*√.43) / ((√.27*.67√.43) + (√.15*.68√.21) + (√.58*.15√.26)) = .56 or 56%. Applying the same calculation to all three mathematics subtests for both genetic and environmental components, genetic factors explain on average 60%, shared environmental factors 26%, and non‐shared environmental factors 14% of the phenotypic correlation between spatial ability and mathematics. Figure 3 summarizes the genetic and environmental origins of the covariation between spatial ability and each of the mathematics subtests. All confidence intervals for the multivariate solution are presented in the supplementary Table S3.

A Cholesky decomposition path diagram, presented in the supplementary Figure S5, shows the partitioning of the variance in the four traits into common and specific influences. The first latent genetic factor A_1_ influences the three mathematical subtests and spatial ability, suggesting that the genetic effects are mostly shared among the measures. The remaining three latent genetic factors show small influences, suggesting very modest genetic influences specific to each measure. The path between Non‐numerical Processes and the spatial composite (.11) indicates that some modest common genetic effects may influence the two traits, independent of other mathematical components. The fourth latent genetic factor A_4_ shows a non‐significant path for the spatial composite, indicating that there is no significant genetic influence on the spatial composite independent of the mathematics measures. Although the influences of shared environmental factors are small, these factors are almost entirely shared among all the measures. The non‐shared environmental influences are largely specific to each trait.

## Discussion

The substantial overlap between spatial and mathematical abilities at age 12 (*r *=* *.43) is driven by both genetic (60%) and environmental factors (40%). Although spatial ability and mathematics are only moderately heritable (*h*^*2*^ = .27 and .43 on average, respectively), the genetic factors contributing to variation in these traits are highly correlated (average genetic *r *=* *.75). This finding leads to the prediction that when genes associated with variation in spatial ability are identified, most will also be associated with mathematical ability. Our results also suggest that, despite reported mean sex differences, the aetiology of individual differences in spatial and mathematical abilities and of the covariation between them is the same for boys and girls.

The mathematical and spatial tests used in this study measured different processes that make up the complex mathematical and spatial domains. For example, Non‐numerical Processes might rely on visual processing to a greater extent than the other two mathematical subtests. Hidden Shapes might require more visual‐attentional processing as compared to the Jigsaws test. However, regardless of the cognitive processes involved, most genetic effects were found to be shared among the four constructs. No independent genetic effects were found on Computation and Knowledge. Small additional genetic effects were present on Non‐numerical Processes, and these effects were partially shared with spatial ability. In other words, these results suggest the presence of some small genetic influences that link spatial ability and non‐numerical mathematical abilities, beyond the general genetic effects on all four constructs. It is possible that these genetic effects are associated with shared demands for visual attention or visuo‐spatial reasoning.

There were no significant genetic effects that were specific to spatial ability once the shared genetic effects with the mathematical measures were taken into account.

The strong genetic overlap between spatial and mathematical ability is in line with previous research showing genetic overlap among a range of cognitive abilities and disabilities – the ‘Generalist Genes’ hypothesis (e.g. Plomin & Kovas, 2005). This research has also shown that genetic effects on diverse cognitive and learning abilities are also associated with variation in general cognitive ability (g) (Davis, Haworth & Plomin, 2009). It is likely that the genetic overlap we observed between spatial and mathematics will also be shared with g; we will specifically explore this link in future investigations. The present investigation focuses on the differential relationships between spatial ability and three different aspects of mathematics. Figure S5 in the Supplementary Materials presents the results of the Cholesky decomposition analyses testing the aetiological differences in the links between three mathematical measures with the spatial ability composite. These results suggest some small specific aetiological links between non‐numerical mathematical processes and spatial ability.

Although the phenotypic relationship between mathematical and spatial abilities is largely due to the same set of pleiotropic genes, about 40% of the overlap between them is attributed to environmental factors. The same shared environments contribute to variation in the three mathematical subtests. The shared environmental correlation between spatial ability and all three mathematical components is very similar (~ .69). Interestingly, both shared and non‐shared environments contribute to the observed overlap between mathematical and spatial abilities, explaining 26% and 14%, respectively. Relevant shared environmental factors might include aspects of the classroom, school, and home experience. Non‐shared environments may include individual specific experiences and perceptions that differ even for identical twins in the same classroom (Asbury, Almeida, Hibel, Harlaar & Plomin, 2008).

Some work has already shown several potentially relevant specific environmental pathways to the link between spatial and mathematical performance. For example, several studies demonstrated positive effects of training on spatial tasks performance (e.g. Baenninger & Newcombe, 1989; Vasta, Knott & Gaze, 1996). If spatial skills, like finger counting and other spatial strategies, are used to bootstrap mathematics, then improving or capitalizing on spatial abilities may improve mathematical learning. The use of visual material in education is on the increase with the hope of improving mathematics and science learning (Phillips *et al*., 2010), with much attention recently devoted to efficient visual communication in STEM fields (see Nature Methods series, Points of View; e.g. Shoresh & Wong, 2012). Intervention studies have shown that improving the ability to think of numbers in spatial terms improves arithmetical skills in typically developing children and those with developmental dyscalculia (e.g. Booth & Siegler, 2008; Kucian, Grond, Rotzer, Henzi, Schönmann, Plangger, Gälli, Martin & von Aster, 2011). Given that early spatial ability has been found to predict expertise in STEM domains, as well as creative and innovative achievements (Wai *et al*., 2009; Kell, Lubinski, Benbow & Steiger, 2013), further research needs to focus on identifying the shared and non‐shared environmental mediators of these associations. These could be used in developing new individualized approaches to fostering spatial ability, potentially supporting further intellectual development in other domains, or vice versa.

As the present investigation has decomposed the co‐variation between mathematical and spatial ability only at one age, it cannot establish any direction of effects. Longitudinal genetically sensitive cross‐lagged designs are necessary in order to examine the presence and nature of any causal links. In addition, more molecular genomic research is needed to identify specific genetic markers and related biological pathways involved in the overlap between mathematical and spatial abilities (Docherty, Davis, Kovas, Meaburn, Dale, Petrill, Schalkwyk & Plomin, 2010).

This study is a step closer towards understanding aetiological links between spatial ability and mathematics. As both are complex domains that involve a wide range of processing, more research is needed to form a more comprehensive picture of these relationships. Our spatial tasks involved manipulation of two‐dimensional objects. Future investigations should include 3‐D mental rotation tasks and other aspects of visuo‐spatial reasoning as these may have different aetiological links to diverse aspects of mathematics. Extending research to samples of different ages and employing longitudinal designs will lead to better understanding of the dynamic nature of mathematic–spatial relationships.

## Supplementary Material

**Figure S1.** Cholesky decomposition of the genetic (1a), shared environmental (1b) and non shared environmental (1c) influences on the 3 mathematical measures and the Jigsaws Test with 95% confidence intervals in brackets, below the estimates. The direct paths (vertical arrows) represent specific genetic and environmental influences, the oblique paths indicate genetic and environmental influences shared among the measures. The comparison between the fit statistics of the multivariate saturated model (‐2LL = 158139.81, df = 38211, BIC = ‐179612.25, ep = 88) and the multivariate ACE model (‐2LL = 91102.13, df = 38265, BIC = ‐247127.25, ep = 34) indicates a good fit of the model to the observed data.
**Figure S2.** Correlated Factor Solution of the model including the Jigsaws Test and the 3 mathematical sub‐tests. The curved arrows represent the genetic (2a), shared environmental (2b) and non‐shared environmental (2c) correlations among the 4 measures. 95% confidence intervals are in brackets, below the estimates. The vertical paths represent the estimates of the heritability (2a), shared (2b) and non‐shared (2c) environmental influences.
**Figure S3.** Cholesky decomposition of the genetic (3a), shared environmental (3b) and non shared environmental (3c) influences on the 3 mathematical measures and the Hidden Shapes Test with 95% confidence intervals in brackets, below the estimates. The direct paths (vertical arrows) represent specific genetic and environmental influences, the oblique paths indicate genetic and environmental influences shared among the measures. The comparison between the fit statistics of the multivariate saturated model (‐2LL = 121703.89, df = 38469, BIC = ‐218328.67, ep = 88) and the multivariate ACE model (‐2LL = 91296.14, df = 38523, BIC = ‐249213.73, ep = 34) indicates a good fit of the model to the observed data.
**Figure S4.** Correlated Factor Solution of the model including the Hidden Shapes Test and the 3 mathematical sub‐tests. The curved arrows represent the genetic (4a), shared environmental (4b) and non‐shared environmental (4c) correlations among the 4 measures. 95% confidence intervals are in brackets, below the estimates. The vertical paths represent the estimates of the heritability (4a), shared (4b) and non‐shared (4c) environmental influences. Small discrepancies between the above estimates the estimates reported in Fig 1S and 2S are due to rounding up the decimal places in the two different model fitting.
**Figure S5.** Cholesky decomposition of the genetic (5a), shared environmental (5b) and non shared environmental (5c) influences on the 3 mathematical measures and the spatial composite. Confidence intervals are in brackets, below the estimates. The direct paths (vertical arrows) represent specific genetic and environmental influences, the oblique paths indicate genetic and environmental influences shared among the measures.
**Table S1.** Intra‐class correlations.
**Table S2.** Univariate sex‐limitation.
**Table S3.** Correlated factor solution.

## References

[desc12138-bib-0001] Asbury, K., Almeida, D., Hibel, J., Harlaar, N., & Plomin, R. (2008). Clones in the classroom: a daily diary study of the nonshared environmental relationship between monozygotic twin differences in school experience and achievement. Twin Research Human Genetics, 11 (6), 586–595 19016614 10.1375/twin.11.6.586 PMC2981605

[desc12138-bib-0002] Astur, R.S., Tropp, J., Sava, S., Constable, R.T., & Markus, E.J. (2004). Sex differences and correlations in a virtual Morris water task, a virtual radial arm maze, and mental rotation. Behavioural Brain Research, 151 (1), 103–115 15084426 10.1016/j.bbr.2003.08.024

[desc12138-bib-0003] Baenninger, M., & Newcombe, N. (1989). The role of experience in spatial test performance: a meta‐analysis. Sex Roles, 20 (5–6), 327–344

[desc12138-bib-0004] Boker, S., Neale, M., Maes, H., Wilde, M., Spiegel, M., Brick, T., Spies, J., Estabrook, R., Kenny, S., Bates, T., Mehta, P., & Fox, J. (2011). OpenMx: an open source extended structural equation modeling framework. Psychometrika, 76 (2), 306–317 23258944 10.1007/s11336-010-9200-6 PMC3525063

[desc12138-bib-0005] Boomsma, D., Busjahn, A., & Peltonen, L. (2002). Classical twin studies and beyond. Nature Reviews Genetics, 3 (11), 872–882 10.1038/nrg932 12415317

[desc12138-bib-0006] Booth, J.L., & Siegler, R.S. (2008). Numerical magnitude representations influence arithmetic learning. Child Development, 79 (4), 1016–1031 18717904 10.1111/j.1467-8624.2008.01173.x

[desc12138-bib-0007] Casey, M.B., Nuttall, R., Pezaris, E., & Benbow, C.P. (1995). The influence of spatial ability on gender differences in mathematics college entrance test scores across diverse samples. Developmental Psychology, 31 (4), 697–705 10.1037//0012-1649.33.4.669 9232382

[desc12138-bib-0008] Davis, O.S., Haworth, C.M., & Plomin, R. (2009). Learning abilities and disabilities: generalist genes in early adolescence. Cognitive Neuropsychiatry, 14 (4–5), 312–331 19634033 10.1080/13546800902797106 PMC2886509

[desc12138-bib-0009] de Hevia, M.D., & Spelke, E.S. (2009). Spontaneous mapping of number and space in adults and young children. Cognition, 110 (2), 198–207 19095223 10.1016/j.cognition.2008.11.003 PMC2705970

[desc12138-bib-0010] Dehaene, S., Bossini, S., & Giraux, P. (1993). The mental representation of parity and number magnitude. Journal of Experimental Psychology: General, 122 (3), 371–396

[desc12138-bib-0011] Docherty, S.J., Davis, O.S.P., Kovas, Y., Meaburn, E.L., Dale, P.S., Petrill, S.A., Schalkwyk, L.C., & Plomin, R. (2010). A genome‐wide association study identifies multiple loci associated with mathematics ability and disability. Genes, Brain and Behavior, 9 (2), 234–247 10.1111/j.1601-183X.2009.00553.x PMC2855870 20039944

[desc12138-bib-0012] Geary, D.C. (1995). Reflections of evolution and culture in children's cognition: implications for mathematical development and instruction. American Psychologist, 50 (1), 24–37 7872578 10.1037//0003-066x.50.1.24

[desc12138-bib-0013] Halpern, D.F. (2000). Sex differences in cognitive abilities. Hillsdale, NJ: Laurence Erlbaum Associates

[desc12138-bib-0014] Haworth, C.M.A., Davis, O.S.P., & Plomin, R. (2013). Twins Early Development Study (TEDS): a genetically sensitive investigation of cognitive and behavioral development from childhood to young adulthood. Twin Research and Human Genetics, 16 (01), 117–125. doi:10.1017/thg.2012.91 23110994 10.1017/thg.2012.91 PMC3817931

[desc12138-bib-0015] Haworth, C.M.A., Harlaar, N., Kovas, Y., Davis, O.S.P., Oliver, B.R., Hayiou‐Thomas, M.E., Frances, J., Busfield, P., McMillan, A., Dale, P.S., & Plomin, R. (2007). Internet cognitive testing of large samples needed in genetic research. Twin Research and Human Genetics, 10 (4), 554–563 17708696 10.1375/twin.10.4.554

[desc12138-bib-0016] Hegarty, M., & Kozhevnikov, M. (1999). Types of visual–spatial representations and mathematical problem solving. Journal of Educational Psychology, 91 (4), 684–689

[desc12138-bib-0017] Hubbard, E.M., Piazza, M., Pinel, P., & Dehaene, S. (2005). Interactions between number and space in parietal cortex. Nature Reviews Neuroscience, 6 (6), 435–448 10.1038/nrn1684 15928716

[desc12138-bib-0018] Hugdahl, K., Thomsen, T., & Ersland, L. (2006). Sex differences in visuo‐spatial processing: an fMRI study of mental rotation. Neuropsychologia, 44 (9), 1575–1583 16678867 10.1016/j.neuropsychologia.2006.01.026

[desc12138-bib-0019] Ito, Y., & Hatta, T. (2004). Spatial structure of quantitative representation of numbers: evidence from the SNARC effect. Memory & Cognition, 32 (4), 662–673 15478760 10.3758/bf03195857

[desc12138-bib-0020] Johnson, E.S. (1984). Sex differences in problem solving. Journal of Educational Psychology, 76 (6), 1359–1371

[desc12138-bib-0021] Kell, H.J., Lubinski, D., Benbow, C.P., & Steiger, J.H. (2013). Creativity and technical innovation: spatial ability's unique role. Psychological Science, 24, 1831–1836 23846718 10.1177/0956797613478615

[desc12138-bib-0022] Kucian, K., Grond, U., Rotzer, S., Henzi, B., Schönmann, C., Plangger, F., Gälli, M., Martin, E., & von Aster, M. (2011). Mental number line training in children with developmental dyscalculia. NeuroImage, 57 (3), 782–795 21295145 10.1016/j.neuroimage.2011.01.070

[desc12138-bib-0023] Lehmann, E.L. (1975). Nonparametrics: Statistical methods based on ranks. San Francisco, CA: Holden‐Day

[desc12138-bib-0024] Levine, S.C., Huttenlocher, J., Taylor, A., & Langrock, A. (1999). Early sex differences in spatial skill. Developmental Psychology, 35, 940–949 10442863 10.1037//0012-1649.35.4.940

[desc12138-bib-0025] Loehlin, J.C. (1996). The Cholesky approach: a cautionary note. Behavior Genetics, 26, 65–69

[desc12138-bib-0026] McGue, M., & Bouchard, T.J. (1984). Adjustment of twin data for the effects of age and sex. Behavior Genetics, 14 (4), 325–343 6542356 10.1007/BF01080045

[desc12138-bib-0027] nferNelson (1994). Mathematics 5–14 series. London: nferNelson Publishing Company

[desc12138-bib-0028] nferNelson (1999). Mathematics 5–14 series. London: nferNelson Publishing Company

[desc12138-bib-0029] nferNelson (2001). Mathematics 5–14 series. London: nferNelson Publishing Company

[desc12138-bib-0030] O'Boyle, M.W., Cunnington, R., Silk, T.J., Vaughan, D., Jackson, G., Syngeniotis, A., & Egan, G.F. (2005). Mathematically gifted male adolescents activate a unique brain network during mental rotation. Cognitive Brain Research, 25 (2), 583–587 16150579 10.1016/j.cogbrainres.2005.08.004

[desc12138-bib-0031] Phillips, L.M., Norris, S.P., & Macnab, J.S. (2010). Visualization in mathematics, reading, and science education. Dordrecht, The Netherlands: Springer

[desc12138-bib-0032] Plomin, R., DeFries, J.C., Knopik, V.S., & Neiderhiser, J.M. (2013). Behavioral genetics (6th edn.). New York: Worth Publishers

[desc12138-bib-0033] Plomin, R., & Kovas, Y. (2005). Generalist genes and learning disabilities. Psychological Bulletin, 131, 592–617 16060804 10.1037/0033-2909.131.4.592

[desc12138-bib-0034] Price, T.S., Freeman, B., Craig, I.W., Petrill, S.A., Ebersole, L., & Plomin, R. (2000). Infant zygosity can be assigned by parental report questionnaire data. Twin Research, 3, 129–133 11035484 10.1375/136905200320565391

[desc12138-bib-0035] R Development Core Team (2011). R: A language and environment for statistical computing. R Foundation for Statistical Computing. Availbale from: http://www.R-project.org/

[desc12138-bib-0036] Raftery, A.E. (1995). Bayesian model selection in social research. Sociological Methodology, 25, 111–163

[desc12138-bib-0037] Rohde, T.E., & Thompson, L.A. (2007). Predicting academic achievement with cognitive ability. Intelligence, 35 (1), 83–92

[desc12138-bib-0038] Rykhlevskaia, E., Uddin, L.Q., Kondos, L., & Menon, V. (2009). Neuroanatomical correlates of developmental dyscalculia: combined evidence from morphometry and tractography. Frontiers in Human Neuroscience, 3, 51 20046827 10.3389/neuro.09.051.2009 PMC2796911

[desc12138-bib-0039] Shoresh, N., & Wong, B. (2012). Data exploration. Nature Methods, 9 (1), 5 22312636 10.1038/nmeth.1829

[desc12138-bib-0040] Shrout, P.E., & Fleiss, J. (1979). Intraclass correlations: uses in assessing rater reliability. Psychological Bulletin, 86 (2), 420–428 18839484 10.1037//0033-2909.86.2.420

[desc12138-bib-0041] Siegler, R.S., & Opfer, J.E. (2003). The development of numeral estimation: evidence for multiple representations of numerical quantity. Psychological Science, 14, 237–243 12741747 10.1111/1467-9280.02438

[desc12138-bib-0042] Smith, P., & Lord, T.R. (2002). Spatial Reasoning 6–14 series. London: NFER Nelson

[desc12138-bib-0043] Thompson, L.A., Detterman, D.K., & Plomin, R. (1991). Associations between cognitive abilities and scholastic achievement: genetic overlap but environmental differences. Psychological Science, 2, 158–165

[desc12138-bib-0044] Tufte, E.R. (2001). The visual display of quantitative information (2nd edn.). Cheshire, CT: Graphics Press

[desc12138-bib-0045] van Garderen, D. (2006). Spatial visualization, visual imagery, and mathematical problem solving of students with varying abilities. Journal of Learning Disabilities, 39 (6), 496–506 17165617 10.1177/00222194060390060201

[desc12138-bib-0046] Vasta, R., Knott, J.A., & Gaze, C.E. (1996). Can spatial training erase the gender differences on the water‐level task? Psychology of Women Quarterly, 20 (4), 549–567

[desc12138-bib-0047] Wai, J., Lubinski, D., & Benbow, C.P. (2009). Spatial ability for STEM domains: aligning over 50 years of cumulative psychological knowledge solidifies its importance. Journal of Educational Psychology, 101 (4), 817–835

[desc12138-bib-0048] Zorzi, M., Priftis, K., & Umiltá, C. (2002). Brain damage: neglect disrupts the mental number line. Nature, 417, 138–139 12000950 10.1038/417138a

