# Supplementary material for: Why do spatial abilities predict mathematical performance?
Source: Dev Sci. 2014 Jan 11;17(3):462–70. doi: 10.1111/desc.12138 (PMC3997754; doi:10.1111/desc.12138)
Supplement: Supplementary file 1 — Figure S1. Cholesky decomposition of the genetic (1a), shared environmental (1b) and non shared environmental (1c) influences on the 3 mathematical measures and the Jigsaws Test with 95% confidence intervals in brackets, below the estimates. The direct paths (vertical arrows) represent specific genetic and environmental influences, the oblique paths indicate genetic and environmental influences shared among the measures. The comparison between the fit statistics of the multivariate saturated model (‐2LL = 158139.81, df = 38211, BIC = ‐179612.25, ep = 88) and the multivariate ACE model (‐2LL = 91102.13, df = 38265, BIC = ‐247127.25, ep = 34) indicates a good fit of the model to the observed data. Figure S2. Correlated Factor Solution of the model including the Jigsaws Test and the 3 mathematical sub‐tests. The curved arrows represent the genetic (2a), shared environmental (2b) and non‐shared environmental (2c) correlations among the 4 measures. 95% confidence intervals are in brackets, below the estimates. The vertical paths represent the estimates of the heritability (2a), shared (2b) and non‐shared (2c) environmental influences. Figure S3. Cholesky decomposition of the genetic (3a), shared environmental (3b) and non shared environmental (3c) influences on the 3 mathematical measures and the Hidden Shapes Test with 95% confidence intervals in brackets, below the estimates. The direct paths (vertical arrows) represent specific genetic and environmental influences, the oblique paths indicate genetic and environmental influences shared among the measures. The comparison between the fit statistics of the multivariate saturated model (‐2LL = 121703.89, df = 38469, BIC = ‐218328.67, ep = 88) and the multivariate ACE model (‐2LL = 91296.14, df = 38523, BIC = ‐249213.73, ep = 34) indicates a good fit of the model to the observed data. Figure S4. Correlated Factor Solution of the model including the Hidden Shapes Test and the 3 mathematical sub‐tests. The curved arrows repre [file desc-17-462-s5.docx]

**Table S1** Intra-class correlations

|  | **mzm** | **mzf** | **dzm** | **dzf** | **dzs** | **dzo** |
| --- | --- | --- | --- | --- | --- | --- |
| ***Spatial Ability*** | .40 ( .33- .46) | .44 ( .39- .49) | .36 ( .39- .43) | .30 ( .23- .36) | .33 ( .28- .37) | .26 ( .21- .31) |
| ***Understanding Numbers*** | .64 ( .60- .68) | .63 ( .59- .66) | .44 ( .37- .50) | .43 ( .37- .48) | .43 ( .39- .47) | .43 ( .38- .47) |
| ***Non-numerical Processes*** | .56 ( .51- .61) | .57 ( .52- .61) | .35 ( .28- .41) | .38 ( .32- .43) | .37 ( .32- .41) | .38 ( .33- .42) |
| ***Computation & Knowledge*** | .60 ( .55- .64) | .60 ( .56- .64) | .41 ( .34- .47) | .41 ( .35- .46) | .41 ( .36- .45) | .35 ( .31- .40) |

mzm = monozygotic male twins; mzf = monozygotic female twins; dzm = dizygotic male twins; dzf = dizygotic female twins dzs = dizygotic same-sex twins; dzo = dizygotic opposite-sex twins

**Table S2** Univariate sex-limitation

|  | ***Δ-2LL (Δdf)*** | ***p*** | ***BIC*** | ***Am*** | ***Af*** | ***Cm*** | ***Cf*** | ***Em*** | ***Ef*** | ***rG*** |
| --- | --- | --- | --- | --- | --- | --- | --- | --- | --- | --- |
| **Spatial Ability** |  |  |  |  |  |  |  |  |  |  |
| *Full sex-limitation* | -- | -- | -28061.35 | .12 (.00-.30) | .28 (.14-.41) | .28 (.14-.14) | .15 (.05-.27) | .59 (.53-.65) | .57 (.52-.62) | .32 (.00-.50) |
| *Null model (no sex effects)* | 29.64 (4) | <.01 | -28064.21 | .26 (.16-.36) | | .16 (.09-.24) | | .58 (.54-.62) | | .50 |
|  |  |  |  |  |  |  |  |  |  |  |
| **Understanding Numbers** |  |  |  |  |  |  |  |  |  |  |
| *Full sex-limitation* | -- | -- | -29757.89 | .42 (.28-.54) | .40 (.29-.52) | .23 (.12-.34) | .23 (.13 -.33) | .36 (.32-.40) | .37 (.34-.40) | .47 (.31-.50) |
| *Null model (no sex effects)* | 8.19 (4) | .08 | -29771.47 | .42 (.35-.49) | | .22 (.16-.27) | | .36 (.34-.39) | | .50 |
|  |  |  |  |  |  |  |  |  |  |  |
| **Non-numerical Processes** |  |  |  |  |  |  |  |  |  |  |
| *Full sex-limitation* | -- | -- | -29558.38 | .41 (.28-.52) | .41 (.29-.51) | .16 (.07-.27) | .16 (.08-.26) | .43 (.39-.48) | .43 (.39-.47) | .50 (.37-.50) |
| *Null model (no sex effects)* | 2.88 (4) | .58 | -29574.62 | .41 (.33-.49) | | .16 (.09-.22) | | .43 (.40-.46) | | .50 |
|  |  |  |  |  |  |  |  |  |  |  |
| **Computation & Knowledge** |  |  |  |  |  |  |  |  |  |  |
| *Full sex-limitation* | -- | -- | -29635.65 | .44 (.31-.58) | .43 (.31-.55) | .17 (.05-.29) | .19 (.08-.29) | .39 (.35-.43) | .39 (.35-.43) | .41 (.24-.50) |
| *Null model (no sex effects)* | 1.98 (4) | .74 | -29652.34 | .47 (.39-.55) | | .14 (.08-.20) | | .39 (.36-.42) | | .50 |
|  |  |  |  |  |  |  |  |  |  |  |

For all four measures, both BIC estimates and overlapping confidence intervals suggested the null model (no sex differences) was the 'best-fitting' model. -2LL = negative twice the log likelihood; df = degrees of freedom; p = p-value; BIC = Bayesian information criterion; Am/ Af = additive genetic variance component, males/ females; Cm/ Cf = shared environmental variance component, males/females; Em/ Ef = non-shared environmental variance component, males/ females; rG = genetic correlation in opposite-sex pairs (fixed at .50 in the Null Model)

**Table S3** Correlated factor solution

|  | ***Spatial Ability -*** | | | ***Understanding Number -*** | | ***Non-numerical Processes -*** |
| --- | --- | --- | --- | --- | --- | --- |
|  | ***Understanding Numbers*** | ***Non-numerical Processes*** | ***Computation & Knowledge*** | ***Non-numerical Processes*** | ***Computation & Knowledge*** | ***Computation and Knowledge*** |
| ***Phenotypic correlation*** | | | | | | |
| ***r_P_*** | .42 (.40 - .44) | .46 (.44 - .48) | .41 (.39 - .42) | .66 (.65 - .67) | .73 (.72 - .74) | .63 (.62 - .64) |
|  | | | | | | |
| ***Mediation of phenotypic correlation*** | | | | | | |
| ***A*** | .54 (.41 - .69) | .66 (.52 - .79) | .59 (.44 - .73) | .53 (.45 - .62) | .60 (.52 - .68) | .58 (.49 - .67) |
| ***C*** | .29 (.17 - .40) | .23 (.12 - .33) | .26 (.14 - .38) | .26 (.19 - .33) | .20 (.14 - .27) | .23 (.15 - .30) |
| ***E*** | .17 (.12 - .22) | .11 (.06 - .16) | .15 (.10 - .21) | .20 (.18 - .23) | .19 (.17 - .22) | .20 (.17 - .23) |
|  | | | | | | |
| ***Genetic, shared and nonshared environmental correlation*** | | | | | | |
| ***r_A_*** | .67 (.61 -.85) | .91 (.74 - 1.00) | .66 (.52 - .83) | .83 (.78 - .91) | .97 (.91 - 1.00) | .82 (.77 - .85) |
| ***r_C_*** | .68 (.45 - .93) | .67 (.43 - .95) | .73 (.45 - .99) | .96 (.83 - 1.00) | .89 (.77 - 1.00) | .98 (.80 - 1.00) |
| ***r_E_*** | .15 (.11 - .20) | .10 (.06 - .15) | .13 (.09 - .18) | .34 (.30 - .38) | .38 (.34 - .42) | .30 (.27 - .34) |
|  |  |  |  |  |  |  |

r_P_ = phenotypic correlation, A = additive genetic factors; C = shared environmental factors; E = non-shared environmental factors; r_A_ = genetic correlation; r_C_ = shared environmental correlation; r_E_ = non-shared environmental correlation; 95% confidence intervals in parentheses

**Figure S1** Cholesky decomposition of the genetic (1a), shared environmental (1b) and non shared environmental (1c) influences on the 3 mathematical measures and the Jigsaws Test with 95% confidence intervals in brackets, below the estimates. The direct paths (vertical arrows) represent specific genetic and environmental influences, the oblique paths indicate genetic and environmental influences shared among the measures. The comparison between the fit statistics of the multivariate saturated model (-2LL = 158139.81, df = 38211, BIC = -179612.25, ep = 88) and the multivariate ACE model (-2LL = 91102.13, df = 38265, BIC = -247127.25, ep = 34) indicates a good fit of the model to the observed data.

**Figure S2** Correlated Factor Solution of the model including the Jigsaws Test and the 3 mathematical sub-tests. The curved arrows represent the genetic (2a), shared environmental (2b) and non-shared environmental (2c) correlations among the 4 measures. 95% confidence intervals are in brackets, below the estimates. The vertical paths represent the estimates of the heritability (2a), shared (2b) and non-shared (2c) environmental influences.

**Figure S3** Cholesky decomposition of the genetic (3a), shared environmental (3b) and non shared environmental (3c) influences on the 3 mathematical measures and the Hidden Shapes Test with 95% confidence intervals in brackets, below the estimates. The direct paths (vertical arrows) represent specific genetic and environmental influences, the oblique paths indicate genetic and environmental influences shared among the measures. The comparison between the fit statistics of the multivariate saturated model (-2LL = 121703.89, df = 38469, BIC = -218328.67, ep = 88) and the multivariate ACE model (-2LL = 91296.14, df = 38523, BIC = -249213.73, ep = 34) indicates a good fit of the model to the observed data.

**Figure S4** Correlated Factor Solution of the model including the Hidden Shapes Test and the 3 mathematical sub-tests. The curved arrows represent the genetic (4a), shared environmental (4b) and non-shared environmental (4c) correlations among the 4 measures. 95% confidence intervals are in brackets, below the estimates. The vertical paths represent the estimates of the heritability (4a), shared (4b) and non-shared (4c) environmental influences. Small discrepancies between the above estimates the estimates reported in Fig 1S and 2S are due to rounding up the decimal places in the two different model fitting.

**Figure S5** Cholesky decomposition of the genetic (5a), shared environmental (5b) and non shared environmental (5c) influences on the 3 mathematical measures and the spatial composite. Confidence intervals are in brackets, below the estimates. The direct paths (vertical arrows) represent specific genetic and environmental influences, the oblique paths indicate genetic and environmental influences shared among the measures.
